# Medication Monitoring for People with Dementia in Care Homes: The Feasibility and Clinical Impact of Nurse-Led Monitoring

**DOI:** 10.1155/2014/843621

**Published:** 2014-02-23

**Authors:** Sue Jordan, Marie Gabe, Louise Newson, Sherrill Snelgrove, Gerwyn Panes, Aldo Picek, Ian T. Russell, Michael Dennis

**Affiliations:** ^1^Department of Nursing, The College of Human and Health Sciences, Swansea University, Singleton Park, Swansea, Wales SA2 8PP, UK; ^2^Fieldbay Ltd., Chestnut House, Tawe Business Village, Swansea Enterprise Park, Swansea SA7 9LA, UK; ^3^The College of Medicine, Swansea University, Singleton Park, Swansea SA2 8PP, UK

## Abstract

*Objectives*. People with dementia are susceptible to adverse effects of medicines. However, they are not always closely monitored. We explored (1) feasibility and (2) clinical impact of nurse-led medication monitoring. *Design*. Feasibility “before-and-after” intervention study. *Setting*. Three care homes in Wales. *Participants*. Eleven service users diagnosed with dementia, taking at least one antipsychotic, antidepressant, or antiepileptic medicine. *Intervention*. West Wales Adverse Drug Reaction (ADR) Profile for Mental Health Medicines. *Outcome Measures*. (1) Feasibility: recruitment, retention, and implementation. (2) Clinical impact: previously undocumented problems identified and ameliorated, as recorded in participants' records before and after introduction of the profile, and one month later. *Results*. Nurses recruited and retained 11 of 29 eligible service users. The profile took 20–25 minutes to implement, caused no harm, and supplemented usual care. Initially, the profile identified previously undocumented problems for all participants (mean 12.7 (SD 4.7)). One month later, some problems had been ameliorated (mean 4.9 (3.6)). Clinical gains included new prescriptions to manage pain (2 participants), psoriasis (1), Parkinsonian symptoms (1), rash (1), dose reduction of benzodiazepines (1), new care plans for oral hygiene, skin problems, and constipation. *Conclusions*. Participants benefited from structured nurse-led medication monitoring. Clinical trials of our ADR Profile are feasible and necessary.

## 1. Background

Patient safety is a priority for healthcare organisations, but there are underlying weaknesses in current practice, particularly medication or drug monitoring for known adverse effects of prescribed drugs [1–4]. Some, 4–6%, of hospital admissions are due to adverse drug reactions (ADRs), most of which are preventable [5, 6]. (An adverse drug reaction is defined as any untoward and unintended response in a patient or investigational subject to a medicinal product which is related to any dose administered [7].) Failure to monitor for common problems, rather than poor prescribing, is responsible for the majority of ADRs [3, 8–13].

Between 25–50% of people with dementia in the UK are prescribed antipsychotic medication [14], but there is local [15] and international variation [16]. For people with dementia, antipsychotics may reduce aggression and psychosis [17–19], particularly amongst those most severely agitated [20]. However, in older people, antipsychotics are associated with increased overall mortality [21–23], worsening cognitive impairment [24], hip fracture [25, 26], diabetes [27], and stroke [14]. Withdrawal of medication reduces falls [28] and improves verbal fluency [18], but aggressive behaviour may return [29], particularly amongst those with the most severe symptoms [30].

Comorbid depression is very common amongst those with dementia, and although antidepressants may have little effect in improving depressive symptoms [31], they may have some benefits in improving agitation [32, 33]. Over one-third of care home residents receive antidepressants [34], sometimes for longer than necessary [35]. However, their use amongst older adults is associated with serious adverse events [36], including serious bleeding [37], paradoxical aggression [38], falls [39], and fractures [25, 40]. In older adults, more subtle adverse effects, such as polyuria, insomnia, or wandering, may predominate, which will only be uncovered by structured ADR monitoring [41, 42].

Some 10–20%, of people with Alzheimer's disease suffer seizures. Older adults are particularly vulnerable to CNS depression and other common adverse effects of antiepileptic drugs (AEDs) [43]. For example, some, 42%, of adults using antiepileptic monotherapy reported depression and 63% reported problems with memory and/or concentration (*n* = 186) [44]. Between 25% and 48% patients discontinue AEDs, depending on the drug (*n* = 1166); reasons include dizziness (lamotrigine, 14.9%), tiredness (levetiracetam, 13.8%), and mood disorders (both drugs 11.7 and 13.8%) [45]. Even those whose epilepsy is well controlled experience subjective complaints, such as problems with cognition >70%, anorexia or nausea or diarrhea >50%, and depression >50% (*n* = 173) [46]. Distinguishing these symptoms from those of dementia may be difficult, but regular monitoring in conjunction with changes in medication regimens may assist recognition of drug-induced symptoms.

The immediate difficulties of managing challenging behaviour, seizures, or depression may be seen as more pressing than possible ADRs or the increase in mortality associated with antipsychotics [21]. Although evidence suggests that nonpharmacological interventions are effective for challenging behaviors, they are not always deployed [47, 48] or available [49]. Prescribers often face a dilemma when asked to advise on management of behavioral problems in people with dementia [50]. While practitioners are warned against prescribing antipsychotics to those with dementia [51, 52], no recommendations on monitoring are offered and systematic reviews offer no consensus on the most effective monitoring strategy [13].

Further research into increased nursing vigilance and improved systems for actively monitoring patients for known adverse effects of prescribed medicines is needed [4, 11, 53–57], and monitoring profiles are ideal [58]. ADR profiles do not replace clinical knowledge and experience, but repackage information into a succinct, formal assessment profile or instrument with potential to address some of the problems relating to failure to monitor prescribed medication [1–4]. Previous work on our structured, nurse-led ADR monitoring profile with 20 adults prescribed antipsychotics indicated that orthostatic hypotension, coupled beats, hypertension, constipation, and inadequate diet were previously undetected [41, 42]. Evidence-based assessment strategies to identify and address ADRs are needed (*cf.* Francis Report, Recommendation 94) [59]. Before undertaking larger studies, we tested the feasibility and clinical impact of our approach to the “ADR problem” [60–64]. Objectives of this pilot were to appraise the logistics of (1) recruitment, retention, and implementation and (2) assessment of clinical benefits of the profile for people with dementia prescribed antipsychotic, antidepressant or antiepileptic medication.

## 2. Methods

### 2.1. Setting

Three private sector nursing home organisations in South West Wales were approached by email, and one responded. The study was undertaken from November 2012 to February 2013 in three homes caring for 81 service users with cognitive deficits and challenging behaviours.

### 2.2. Participants

**Figure 1:**
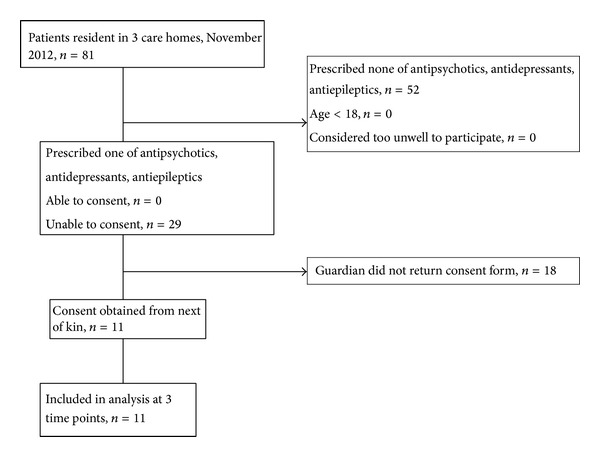
Participant flow diagram.

Service users were assessed for eligibility and approached by their nurses. Inclusion criteria were: resident at the care home; diagnosed with dementia, currently taking antipsychotics and/or antiepileptics and/or antidepressants, and willing and able to give informed, signed consent themselves, or where capacity was lacking, a guardian was willing to give informed, signed consent. We excluded service users aged <18 and those considered by their nurses too unwell to participate. Recruitment and follow-up are summarized in Figure 1. This was a feasibility study, and no sample size was calculated [65, 66].

### 2.3. Design

This prospective “before and after” record review explored the feasibility, implementation, and clinical gains from nurse-led medication monitoring using the West Wales ADR Profile for Mental Health Medicines on two occasions, one month apart. Service users' records were reviewed on three occasions: (1) before introduction of the profile, (2) after the first use of the profile, and (3) after the second use of the profile one month later.

### 2.4. Intervention

The West Wales ADR Profile for Mental Health Medicines offers a comprehensive, structured, adverse drug reaction (ADR) template (see Supplementary Appendix  1 in Supplementary Martial, available online at http://dx.doi.org/10.1155/2014/843621). It aims to alleviate any problems of underreporting of ADRs and facilitate shared decision-making with service users and within the multidisciplinary team [67].

The ADR Profile links identification with actions for 80 problems potentially associated with antipsychotic, antidepressant, or antiepileptic medicines. It contains five sections: vital signs, observations, directed questions, health promotion, and care planning. The first three sections are designed to be passed to prescribers ahead of medication reviews, with problems highlighted. Health promotion and care planning sections serve to direct nursing care. The profile was developed from earlier versions [60–64], incorporating ADRs documented in formularies [52, 68] and manufacturers' literature. Interrater reliability for the items' kappa values ranged from 0.44 to 1.00, with clinical observations having generally lower values [61]. The profile was administered by nurses during routine care. This involved observing or questioning service users and reviewing care plans. Key stakeholders, including service user representatives and clinicians, were involved before, during, and after the study, providing their perspective and input on the design of the study and the ADR profile.

### 2.5. Outcomes

1. Feasibility outcomes for future projects [69] are
  - recruitment and retention of institutions and service users,
  - nurses' compliance with medication monitoring, both recording and addressing problems identified,
  - nurses' reports of implementation of the ADR Profile.
2. Clinical gains and estimate of the value of pursuing this strategy for harm reduction are
  - problems newly identified and addressed within 1 month (Table 1),
  - changes in medication regimens,
  - record of functional status or “what the service user can do,” using the Crichton Royal Behaviour Rating Scale (CRBRS); a score of 38 indicates maximum dependency [70–72]. This measure is administered monthly to all service users in the care homes, as a component of normal care.

### 2.6. Data Collection

Feedback on the ADR Profile, its utility, and the potential for clinical gain were sought from nurses in short semistructured interviews at the start and end of the project. They are reported to inform future work.

Service users' records were reviewed before intervention (round 1), after first completion of the profile (round 2), and after second, follow-up, completion of the profile (round 3). We extracted data from service users' case notes and completed profiles to identify (a) evidence of previous medication monitoring, (b) whether information on the ADR Profile had already been captured, (c) actions undertaken following administration of the ADR Profile, and (d) clinical gains at follow up. We noted the following:

- number and nature of problems documented as present, actioned or discussed with prescribers with and without the profile,
- follow-up actions and change in care plans,
- changes in prescription regimens as documented in administration records and referral letters,
- record of functional status, the CRBRS,
- other evidence of clinical change and endpoints.

### 2.7. Analysis

Data were entered into IBM SPSS statistics v.19 and described. Each profile item represented a single variable. Problems noted and actions documented as taken were summed. Problems not previously documented were enumerated. Differences between before (without) and after (with) the profile and between first and second administration were described. CRBRS scores were tentatively explored using one-way repeated measures ANOVA.

Nurses' views were recorded and summarised to develop the WWADR Profile and inform the feasibility of larger studies.

### 2.8. Ethics

Approval for the study was granted by the SW Wales NHS Research Ethics committee (reference: 12/WA/0311, 23.10.2012). The University's College of Human and Health Science Research Ethics Committee granted approval for interviews with nurses working in the private sector. Service users' or guardians' written consent for researchers to review service users' notes was sought by qualified nurses, who were familiar with the Mental Capacity Act (2005), and employed by the care homes. Service users' General Practitioners (GPs) were informed of the project by letter. It was agreed that the project posed no physical risk to service users or staff. Usual standard care was delivered throughout.

## 3. Results

### 3.1. Feasibility

#### 3.1.1. Recruitment and Retention

Of 81 residents in three care homes, 29 met the inclusion criteria, and 11 guardians gave written consent to participation. All participants were unable to consent for themselves. There was no loss to follow up (Figure 1). Interval variables were normally distributed (Shapiro-Wilk *P* > 0.1 for all variables). At baseline, service users' mean (SD) age was 71.9 (16.4) years, range 39–96; 3 were male, as were 9 of 29 eligible participants. The mean (SD) number of prescribed medicines was 11.0 (5.5), range 3–22: 6 participants received antipsychotics, 6 antidepressants, and 8 antiepileptics. Indications for prescriptions were not recorded in nursing notes. Many medication regimens had been initiated prior to arrival at the care home. No alternative medication monitoring instruments, such as the Liverpool University Neuroleptic Side Effect Rating Scale [73], were found in the service users' documentation.

The severe cognitive deficits of many participants necessitated proxy respondents for several items and the assessment of overall functioning [67]. Some questions required verbal communication, for example, the presence of tinnitus or “pins and needles,” but this was impossible for some service users. Most items on the profile were completed with all service users. One service user was immobile and unable to stand; therefore, items relating to gait, balance, and postural hypotension were inapplicable. Girth measurement, questions on sweating, libido, and balance assessment were completed by <9 respondents. Nine to 11 service users completed all other items. Laboratory and ECG results were not recorded in the care homes' notes.

#### 3.1.2. Implementation of the ADR Profile

Nurses indicated that engagement with relatives to gain written consent took time and was not always successful. Some relatives were unable to understand that the study was noninvasive and did not involve administration of new medicines. Others simply forgot to return the consent form, and many were facing personal difficulties, rendering return of a consent form a low priority. Burdening elderly, frail relatives with long information sheets when the project was directed at nursing care and enhancing documentation seemed inappropriate.

All six qualified nurses completing the profiles agreed that it caused no harm and took 20–25 minutes to complete, including assessment of vital signs and girth. Time taken depended on service users' problems and nurses' familiarity with the profile. With 80 questions, the profile was considered comprehensive and included some issues not routinely monitored, for example, bleeding, hair loss, and oral care. The list of 80 items was longer than could easily be memorised. Therefore, the profile ensured ADRs were not overlooked and reinforced educational initiatives. Nurses believed that although service users benefited from structured nurse-led medication monitoring, their underlying conditions remained unchanged. The profile is now completed in conjunction with other regular nurse-led monitoring, for example, for pressure sores, and discussed with prescribers.

### 3.2. Clinical Gains

#### 3.2.1. Problems Identified

On first administration, the WWADR Profile identified previously undocumented problems for all service users: mean 12.7 (SD 4.7), 95% CI 9.6–15.9, range 8–22 (Table 1). Some problems, such as abnormal movements (4), postural hypotension (2), pain (2), and fluid intake, were recorded and monitored for the first time. On second administration of the profile 1 month later, further new problems were identified mean: 4.7 (SD 5.0), 95% CI 1.4–8.0, range 0–18. Some of these had probably arisen in the intervening period, for example, tachycardia, leaving meals unfinished, and missing doses of medicines. Others were unlikely to have arisen in the last month, and may have been overlooked at first use of the profile due to familiarity, for example, posture or extensive preexisting documentation of the problem, for example, convulsions.

#### 3.2.2. Problems Addressed

When the ADR profiles were readministered at follow-up 1 month later, all service users had had at least one problem ameliorated: mean 4.9 (SD 3.6), 95% CI 2.5–7.3, range 1–11 (Table 1). Problems marked as addressed included access to dentist (*n* = 5) (one service user needed a filling), constipation (*n* = 2), and fluid intake normalised (*n* = 4). New care plans were in place for the following:

- oral hygiene (*n* = 2),
- risk of dehydration due to low fluid intake (*n* = 2),
- skin care (*n* = 2),
- constipation (*n* = 1),
- postural aids (*n* = 1).

Other problems, such as aggression and cognitive decline, were more intractable. Some problems, such as intake of sugary drinks, remained.

Three new medicines were prescribed at follow-up:

- creams to manage psoriasis, however, the rash remained at follow up,
- terbinafine 1% *mane* to treat a rash,
- a referral to the Parkinson's service and a trial of co-beneldopa for movement, posture, and gait problems.

Two therapeutic regimens were revised, in response to problems found:

- increased analgesia,
- increased and regularised administration of analgesic creams for arthritic pain in the knee or leg. Abnormal posture associated with arthritis was noted to have ameliorated at follow up.

One service user was noted to be oversedated, and benzodiazepine prescribing was reduced. Sedation was no longer indicated as a problem at follow-up.

CRBRS scores indicated high dependency and remained largely unchanged throughout the study, despite amelioration of ADR-related problems. Mean (SD) scores and ranges in the 3 rounds were 33.5 (3.1), 29–23; 33.4 (2.6), 30–37; and 33.9 (2.6), 30–37. One-way repeated measures ANOVA indicated no significant differences between the 3 rounds of data collection.

## 4. Discussion

Recruiting and retaining participants and implementing medication monitoring in care homes is feasible. Implementation of the ADR Profile identified unsuspected clinical problems and enhanced care for all service users. On follow-up some (mean 4.9, range 1–11) problems had resolved; dentists' visits had been arranged for five service users, fluid intake had improved for four, five new therapeutic regimens had been introduced, one benzodiazepine regimen had been reduced, and new care plans were in place covering oral hygiene, psoriasis, skin integrity, postural aids, and constipation. Although service users' underlying dependency, as measured by the CRBRS, remained unchanged, some problems had been addressed, including over-sedation, constipation and fluid intake.

### 4.1. Strengths and Weaknesses

This was a feasibility study, without a comparator or “control” group, typical of adverse event research [74]. Low numbers precluded inferential analyses. Duration of follow-up was suboptimal, as in large trials of ADR initiatives [13], reflecting available resources. As in all uncontrolled adverse event studies, signs and symptoms may have been related to underlying conditions, concurrent therapy, or spontaneous events [74], and we cannot infer causation of the problems identified. However, to service users, amelioration may be more important than aetiology, and problem identification is a necessary prerequisite to management. We did not assess the “appropriateness” of prescribing [75] or the severity of problems identified; however, participating clinicians considered many of these (56/141, 40%) merited intervention.

We cannot assume that respondents and response patterns are representative of other organisations. Care home residents are often more frail and vulnerable than community dwellers, and their cognitive impairment may restrict their ability to communicate medicine-related problems [76]. However, this is the third clinical area to indicate that nurse-led monitoring detects previously unsuspected problems efficiently [41, 42, 64]. The mean number of medicines per participant (11.0, range 3–22) was higher than previously reported [77], but in line with upward trends [13]. South West Wales may not be untypical of the UK; local hospitals performed well in UK-wide audit of medication monitoring [61, 78, 79].

All research is vulnerable to bias and confounding [80]. We are particularly concerned with diagnostic or “exposure suspicion” bias, that is, knowledge of the patients' prior exposure to a drug or disease influencing recording of signs or symptoms [81, pp. 55-56]. This might have increased the numbers of problems ticked on the profiles had nurses been mindful of service users' histories. However, any spuriously identified problems would probably not have been addressed.

### 4.2. Future Research

The benefits to service users indicate that this work should be developed, using methodological insights gained. Recruitment cannot be predicted by hypothetical discussions [82]. Using the inclusion criteria and settings and procedures planned for larger studies [69], 11/29 (34%) eligible service users were recruited. Anecdotally, recruitment favoured better educated families [83]. Whilst this nonresponse bias [84] potentially limits generalisation of findings, we have no reason to speculate that sociodemographic differences would be reflected in care received. Taken with nurses' reports that the need for written consent excluded many potential participants, the absence of contact between researchers and service users, the difficulties in obtaining consent in this field [76], and evidence that nurse-led medication monitoring does not cause harm, it would seem appropriate to opt for verbal, rather than written, consent in further study of ADR profiles. Administration of the ADR Profile conferred no greater risk than a clinical examination [85, page  34].

Guidelines, computerised reminders, academic detailing, safety initiatives, and medication reviews improve the process of care; however, evidence of improved outcomes is scarce [86–90]. Global outcome measures require further methodological investigation; there may be no consensus over minimally important differences, and scoring and reliance on statistical significance may obfuscate any clinical improvements [67]. Benefits of ADR profiling may be confined to the specific problems identified, such as pain, stiffness, dehydration, and oral care, and not reflected in global measures. Cochrane reviews (including 1186 and 7653 patients) suggest that mortality and readmission to hospital may be insufficiently sensitive to medication review [13, 91]. Similarly, instruments, such as the CRBRS or Bartel's index [72, 92] or quality of life or behaviour measures [93], while useful when comparing populations, may be insufficiently responsive to subtle but important changes, such as improved oral care, rendering them less suitable for clinical trials.

One of 11 service users had medication reduced, while 5 had new medicines prescribed. Underprescribing of beneficial medicines was more easily addressed than overprescribing; the latter involves designating a long-term medicine “inappropriate” [77, 93]. Therefore, “number of prescription items per participant” appeared a crude measure of change, and would not be a reliable outcome measure [94, 95].

### 4.3. Clinical Implications

#### 4.3.1. The ADR Problem

All service users had previously unrecognized ADRs and at least one problem ameliorated [41, 42, 64]. Clinicians were aware of appropriate strategies, and the profile guidelines offered suggestions. As in larger cohorts in primary care [96] and long-term care [9], SSRIs were frequently prescribed [96, 97]. The true prevalence of ADRs is unknown [11], but people with dementia are very vulnerable [98]. The US Office of the Inspector General [99, page  ii] reported that nursing homes' patients “are experiencing numerous adverse reactions as a result of potentially inappropriate prescribing and inadequate administration or monitoring of the usage of medications”; antipsychotics and antidepressants were frequently implicated. The problems described reflect our findings: constipation, falls, and urinary incontinence.

Nurse-led medication monitoring has potential to mitigate the widespread problem of underreporting of ADRs, as evidenced by others: 95% of serious ADRs in hospitals were not reported [100]; none of 26 adverse events detected by record review were reported on critical incident reports [101]; seven (2.2%) of 325 ADRs identified by database review were spontaneously reported [102]; and 9% (376/4211) of adverse events were reported to the UK Committee for Safety of Medicines, by yellow cards [103]. Structured monitoring may also address the disparity and low interrater reliability between doctors' and patients' identification of adverse effects of antipsychotics [104].

Published “side effect” instruments only consider antipsychotics; this would have excluded 13 of our 29 potential participants; antidepressants and antiepileptics can also have important adverse effects. Some instruments rely on patients' self-report [73] or are less than comprehensive [61]. 

### 4.4. Successful Monitoring

Successful monitoring, like effective screening, should be accurate, simple, thorough, target-based, and capable of detecting insidious or long-term harm and should improve clinical outcomes [105]. Effective pharmacotherapy includes reliable assessment of drug effects [90], particularly in UK care homes [106]. Paper-based reminders improve the process of care, particularly when they demand responses from professionals [89]. Checklists have reduced adverse events in other areas of health care [107, 108]. Of the ten cases, nine fatal, of substandard care by several professions reported by the Health Service Commissioner (2011), four relate to failure to assess elderly patients, including one failure to monitor prescribed olanzapine [109].

Current formal pharmacovigilance strategies to detect and record ADRs are inadequate, either due to uncertainties in coding systems or loss of power due to intricate subdivisions in signs and symptoms [110]. Psychiatrists' consultations are usually informal and driven by the professional; this avoidance of detailed directed questioning allows any reports of ADRs to be overlooked [111, 112]. Therefore, administration of the ADR Profile by nurses prior to doctors' appointments has potential to bridge this hiatus in care. Not all problems identified were actioned, for example, due to clinical judgment or lack of time, and this might be improved with closer medical involvement.

ADR profiles should not duplicate existing documentation or increase professionals' bureaucratic burden [113]. Where nurses undertake tasks outside traditional nursing roles, time [114] and educational preparation [115] may be perceived barriers. Any change needs to be seen as beneficial, resourced, and achievable [3]. To allay concerns regarding the necessary investment in time and learning, evidence is needed for the clinical effectiveness of ADR profiles in identifying and ameliorating the burdens of treatment [116]. Healthcare professionals, particularly doctors, are unlikely to accept implementation of any monitoring procedures without evidence from clinical trials [117, 118].

## 5. Conclusions and Implications

We highlighted opportunities to improve medication management, increase recognition of ADRs, and augment communication between prescribers and nurses. The 20–25 minutes of nurses' time to administer the profile was worthwhile. Taken with previous work [41, 42, 64], this study suggests that ADR profiles have potential to address unmet clinical need [58]. Given the distress caused by suboptimal oral care or analgesia, the potential health service costs of urgent dental treatment or falls due to high doses of benzodiazepines, and the small investment in nursing time needed to complete ADR profiles, our strategy merits further investigation.

Service users are rarely monitored for possible adverse reactions to their medicines, and there is no consensus on how to do this [1–4, 8–13]. Absence of a standard system for long-term monitoring of ADRs is a barrier to implementation [119], which our research programme aims to address. Implementation of simple, noninvasive, and affordable ADR profiles would expedite some recommendations from recent inquiry into UK health care failings: enhanced health care information, transparency, enhanced interdisciplinary teamwork, easily accessible and systematic recording of routine observations, and medication management involving frequent checking to minimize drug errors [59]. Incorporation of ADR profiling into policy initiatives would rectify current weaknesses in practice [1–4, 59], but requires evidence from multicentre clinical trials [120].

## Summary

(i) To our knowledge, this is the first nurse-led study to suggest how the impact of adverse drug reactions (ADRs) in people with dementia might be ameliorated using existing resources. (ii) Without structured, formalised ADR monitoring profiles, problems putatively associated with prescribed medicines are easily overlooked. (iii) The clinical changes resulting from nurse-led medication monitoring were important but not reflected in global outcome measures. (iv) Obtaining signed consent for noninvasive research from relatives of people with dementia, who were themselves often unwell, reduced sample size. (v) Larger trials are feasible and are needed to quantify the benefits of structured nurse-led medication monitoring before this intervention is recommended for adoption.

## Supplementary Material

The supplementary material offers the version of the West Wales ADR Profile for Mental Health Medicines used in this study.

## Figures and Tables

**Table 1:** Problems identified for the first time by the WWADR profile and problems noted as ameliorated at follow-up (*n* = 11).

Problem	New problem at first use	Ameliorated at follow-up
Postural hypotension	2	1
Girth	1	0
Hand tremor	3	2
Feet shuffling	2	0
Abnormal movements	4	2
Posture	6	2
Gait	6	3
Balance/coordination	8	2
Bleeding or bruising	1	1
Cognitive decline	5	3
Concentration declining	5	3
Convulsions	3	2
Self-harm/violence	1	1
Irritability or aggression	1	0
Behaviour	2	1
Restlessness or pacing	3	0
Sleep problems/insomnia	4	3
Sleep/sedation	5	2
Confusion	3	1
Low energy, weakness, fatigue, apathy	1	1
Mood fluctuations	5	2
Agitation, anxiety, nervousness	4	0
Hyperactivity	1	0
Urination	1	1
Constipation	4	2
Rash (+/− itching)	1	0
Swelling/oedema, particularly pressure areas	2	1
Sweating, particularly pressure areas	1	0
“Snacking” between meals	2	2
Drinking less than 1 pint of milk per day	10	3
Drinking less than 2 litres per day	5	4
Access to sugar free drinks	10	3
Swallowing	1	0
Indigestion/heartburn	3	2
Teeth/dentures/mouth	1	0
Dentist not seen in last 12 months	9	5
Optician not seen in last 12 months	3	1
Dark glasses not worn in bright sunlight	10	0
Pain	2	0

Total	141	56
