# Supplementary material for: Medication Monitoring for People with Dementia in Care Homes: The Feasibility and Clinical Impact of Nurse-Led Monitoring
Source: ScientificWorldJournal. 2014 Feb 23;2014:843621. doi: 10.1155/2014/843621 (PMC3951004; doi:10.1155/2014/843621)
Supplement: Supplementary file 1 — The supplementary material offers the version of the West Wales ADR Profile for Mental Health Medicines used in this study. [file 843621.f1.docx]

## sub 8.1.14 Appendix 1

## West Wales Adverse Drug Reaction Profile for Mental Health Medicines

version V

## Patient ID …………… Date _ _/_ _/_ _

**Completed** ________ ____________ **Profession:** _________________

**Authorised by** _____________________ **Profession:** _________________

Please complete pages 1-4. Circle to identify the presence or absence of a problem. Please provide information if the problem is worsening. Please inform prescriber of problems.

Information on action(s) taken to be recorded and care plan to be formulated on page 5. Guidelines are available from authors on request. This is a profile: scoring is not required.

If the problem has worsened since the last review, highlight ‘worse’

| **Section One. Vital signs** | | | **Problem** | | **Actions** |
| --- | --- | --- | --- | --- | --- |
| Heart rate | bpm | | no / yes | |  |
| Irregular rhythm | | | no / yes | |  |
| BP lying/ sitting | / mmHg | | no / yes | |  |
| BP standing | / mmHg | | no / yes | |  |
| Weight / BMI | Kg kg/m^2^ | | no / yes | |  |
| Change since last recording | | | loss / gain | |  |
| Girth | cm | | no / yes | |  |
| Change since last recording | | | decrease /increase | |  |
| Temperature | °C | | no / yes | |  |
| **Section Two. Observations of problems** | | | | | **Actions** |
| Hand tremor | | no / yes / worse | |  | |
| Tongue tremor | | no / yes / worse | |  | |
| Feet shuffling | | no / yes / worse | |  | |
| Abnormal movements | | no / yes / worse | |  | |
| Posture abnormal | | no / yes / worse | |  | |
| Gait abnormal on walking | | no / yes / worse | |  | |
| Balance abnormal/ co-ordination poor & interferes with ADLs | | no / yes / worse | |  | |
| Bleeding or bruising | | no / yes / worse | |  | |
| Feeling the cold | | no / yes / worse | |  | |
| Cognitive decline | | no / yes / worse | | Last documentation date | |
|  | | no / yes / worse | |  |  |
| Concentration declining | | no / yes / worse | |  |  |

| **Section Three. Reports of potential problems** | | | | **Actions** | | | |
| --- | --- | --- | --- | --- | --- | --- | --- |
| Convulsions | no / yes / worse |  | | | | |  |
| Self-harm or violence | no / yes / worse |  | | | | |  |
| Irritability or aggression | no / yes / worse |  | | | | |  |
| Mania | no / yes / worse |  | | | | |  |
| Behaviour problems | no / yes / worse |  | | | | |  |
| Restlessness or pacing | no / yes / worse |  | | | | |  |
| Falls / Dizziness | no / yes / worse |  | | | | |  |
| Sleep problems/ insomnia | no / yes / worse |  | | | | |  |
| Sleep / sedation | no / yes / worse |  | | | | |  |
| Confusion | no / yes / worse |  | | | | |  |
| Low energy, weakness, fatigue, apathy | no / yes / worse |  | | | | |  |
| Mood fluctuations | no / yes / worse |  | | | | |  |
| Agitation, anxiety, nervousness | no / yes / worse |  | | | | |  |
| Hyperactivity | no / yes / worse |  | | | | |  |
| Panic attacks | no / yes / worse |  | | | | |  |
| Hallucinations, vivid dreams | no / yes / worse |  | | | | |  |
| Headache, migraine | no / yes / worse |  | | | | |  |
| Tinnitus/hearing problems | no / yes / worse |  | | | | |  |
| Tingling/pins & needles | no / yes / worse |  | | | | |  |
| Urination | no / yes / worse |  | | | | |  |
| Reproductive system e.g.  Change in libido | no / yes / worse |  | | | | |  |
| Chest pain | no / yes / worse |  | | | | |  |
| Palpitations | no / yes / worse |  | | | | |  |
| Short of breath | no / yes / worse |  | | | | |  |
| Hypersalivation/  respiratory tract infection | no / yes / worse |  | | | | |  |
| Nausea / vomiting | no / yes / worse |  | | | | |  |
| Appetite/ taste changes | no / yes / worse |  | | | | |  |
| Bowel control/ diarrhoea | no / yes / worse |  | | | | |  |
| Constipation | no / yes / worse |  | | | | |  |
| Rash (+/- itching) | no / yes / worse |  | | | | |  |
| Swelling/ oedema / pressure areas | no / yes / worse |  | | | | |  |
| Sweating / pressure areas |  |  | | | | |  |
| Injection site e.g. pain | no / yes / worse | NA | | | | |  |
| Other problems | Actions taken. Add details to p.4 | | | | |  |  |
| **Section Four: Health promotion** | | | | | **Actions** | | |
| Eating at least 1 cooked meal daily? | | | yes/no | |  | | |
| Missing any meals or leave them unfinished more than once a day? | | | no/yes | |  | | |
| ‘Snacking’ or eating between meals? | | | no/yes | | Examples: | | |
| Drinking 1 pint or more of milk or soya milk per day? This includes milk added to cereal & hot drinks | | | yes/no | |  | | |
| Vitamin D intake adequate (time in sunlight, eats oily fish) | | | yes/no | |  | | |
| Eating fruit or vegetables every day? | | | yes/no | |  | | |
| Drinking more than 2 litres, or 6-8 cups, per day? This includes water, tea, coffee or squash | | | yes/no | |  | | |
| Are drinks sugar free? | | | yes/no | |  | | |
| Swallowing difficulties | | | No/yes | |  | | |
| Indigestion or heartburn | | | no/yes | |  | | |
| Medicines used for this? | | | yes / no | |  | | |
| Dentists | | | | | | | |
| Problems with teeth or dentures | | | no/yes | |  | | |
| Dry mouth | | | no/yes | |  | | |
| Halitosis | | | no/yes | |  | | |
| Dentist seen in last 12 months | | | yes / no | |  | | |
| Smoking | | | no/yes | | Number per day: | | |
| Opticians | | | | | | | |
| Vision problems or dry eyes | | | no / yes | |  | | |
| Optician seen in last 12 months | | | yes / no | |  | | |
| **Skin problems** | | | | | | | |
| Is sunscreen available? | | | yes / no | |  | | |
| Is it applied evenly? | | | yes / no | |  | | |
| Is the sunscreen adequate? | | | yes / no | |  | | |
| Dark glasses worn in bright sunlight? | | | yes / no | |  | | |
| Hair loss | | | no / yes | |  | | |
| Acne or *Herpes simplex* reactivation | | | no / yes | |  | | |
| Advice sought for this? | | | yes / no | |  | | |
| **Pain** | | | | | | | |
| Any pain? e.g. joint pain, headache | | | no / yes | |  | | |
| Medicines used for this? | | | yes / no | |  | | |
| **Medicines administration** including patient refusal Use these 3 questions | | | | | | | |
| Regular medication taken at the same time each day? | | | yes/no | |  | | |
| More than 2 doses of prescribed medication missed over any period of seven days in the last month? | | | no/yes | |  | | |
| Medication taken with or immediately after food? | | | yes/no | |  | | |

| Section six. Action plan. Please record intended actions and formulate a care plan based on problems identified. Please see guidelines for further information (page 5) | |
| --- | --- |
| Problem | **Care plan** |

Continue on separate sheet if necessary and append.**Current medication regimen**

Information can be gathered by researchers or added by care staff, at the discretion of care staff.

| Drug | Dose | Taken at (Times of day) | Date started | Prescriber | Date last modified |
| --- | --- | --- | --- | --- | --- |
|  |  |  |  | GP/consultant |  |
|  |  |  |  | GP/consultant |  |
|  |  |  |  | GP/consultant |  |
|  |  |  |  | GP/consultant |  |
|  |  |  |  | GP/consultant |  |
|  |  |  |  | GP/consultant |  |
|  |  |  |  | GP/consultant |  |

*Bibliography used to compile this version of the WWADR Profile*

ABPI Compendium of Data sheets and Summaries of Product Characteristics (updated yearly) Datapharm Publications Ltd : London. (Pharmacy Dept) http://www.medicines.org.uk/

Aronson JK (ed.) 2006 Meyler's Side Effects of Drugs: The International Encyclopedia of Adverse Drug Reactions and Interactions Copyright © 2006 Elsevier B.V. Editor:  J.K. Aronson
Shortcut URL to this page: <http://www.sciencedirect.com/science/referenceworks/0444510052>

Baxter K. (2011) *Stockley’s Drug Interactions*. Blackwell Science, Oxford. 7^th^ edition

Berman A., Snyder S., Kozier B., Erb G. 2008 *Kozier & Erb’s fundamentals of Nursing.* Pearson, NJ. 8^th^ ed.

BNF (British National Formulary) CURRENT EDITION British Medical Association and the Royal Pharmaceutical Society of Great Britain, London. http://www.bnf.org/bnf/

Brunton L. Lazo J., Parker K. (eds.) 2006 Goodman & Gilman’s: The Pharmacological Basis of therapeutics. New York, McGraw-Hill, 11^th^ edition.

Cunningham Owens DG. 1999 *A Guide to the Extrapyramidal Side-Effects of Antipsychotic Drugs.*  Cambridge University Press

Doran C. 2003 *Prescribing mental health medication.*  Routledge, London

Jordan S., Knight J., Pointon D. 2004 Monitoring Adverse Drug Reactions: Scales, Profiles and Checklists. *International Nursing Review.* 51, 208-221

Jordan S. 2008 *The Prescription Drug Guide for Nurses*. Open University Press, McGraw-Hill, Maidenhead.

Kane JM (ed.) 1999 *Managing the Side Effects of Drug Therapy in Schizophrenia*. London: Science Press

[Ohlsen](http://catalogue.informahealthcare.com/pjbp/search.htm?f=editor&q=Ohlsen%20Ruth) R., [Pilowsky](http://catalogue.informahealthcare.com/pjbp/search.htm?f=editor&q=Pilowsky%20Lyn) L., [Smith](http://catalogue.informahealthcare.com/pjbp/search.htm?f=editor&q=Smith%20Shubulade) S., Taylor D. (Eds) 2003 Maudsley Antipsychotic Medication Review Service Guidelines. Informa healthcare, London

[Ohlsen](http://catalogue.informahealthcare.com/pjbp/search.htm?f=editor&q=Ohlsen%20Ruth) R., [Pilowsky](http://catalogue.informahealthcare.com/pjbp/search.htm?f=editor&q=Pilowsky%20Lyn) L., [Smith](http://catalogue.informahealthcare.com/pjbp/search.htm?f=editor&q=Smith%20Shubulade) S., Taylor D. (Eds) 2003 Maudsley Antipsychotic Medication Review Service Guidelines. Informa healthcare, London

Smith S., Duell D., Martin B. 2008 *Clinical Nursing Skills: Basic to Advanced Skills*. Pearson, Prentice Hall, New Jersey. 7^th^ edtn

*Sockalingam S. et al 2007 Clozapine-induced hypersalivation. Canadian Journal of Psychiatry. 52,6,377-84*

[Taylor](http://catalogue.informahealthcare.com/pjbp/search.htm?f=editor&q=Taylor%20David) D., [Paton](http://catalogue.informahealthcare.com/pjbp/search.htm?f=editor&q=Paton%20Carol) C., [Kerwin](http://catalogue.informahealthcare.com/pjbp/search.htm?f=editor&q=Kerwin%20Robert) R. 2007 The Maudsley Prescribing Guidelines, Ninth Edition, Informa healthcare, London
